# Determining the cause of intrauterine fetal death in monochorionic twins: A case report

**DOI:** 10.3389/fmed.2022.1055275

**Published:** 2023-01-04

**Authors:** Anxia Xie, Yan Cui, Gang Luo, Xianxia Chen, Xuecheng Zhang, Jie Han, Li Tong, Yanming Ren, Xiaoxing Wei

**Affiliations:** ^1^Research Center for High Altitude Medicine, Qinghai University, Xining, China; ^2^Medical College, Qinghai University, Xining, China; ^3^Department of Obstetrics, Qinghai Provincial People’s Hospital, Xining, China; ^4^Department of Gynecology, Qinghai Provincial People’s Hospital, Xining, China; ^5^Department of Anaesthesiology and Perioperative Medicine, Xijing Hospital, The Fourth Military Medical University, Xi’an, China; ^6^Department of Ultrasonography, Qinghai Provincial People’s Hospital, Xining, China; ^7^Department of Pathology, Qinghai Provincial People’s Hospital, Xining, China; ^8^Qinghai Provincial Key Laboratory of Traditional Chinese Medicine Research for Glucolipid Metabolic Diseases, Xining, China

**Keywords:** fetal death, double-embryo transfer, monochorionic dizygotic (MCDZ) twins, partial trisomy 9, vascular anastomoses, case report

## Abstract

**Background:**

Determining the cause of intrauterine fetal death is essential for patients to manage their next pregnancy. However, in the majority of cases of fetal death, the cause remains unexplained despite comprehensive evaluation, especially in the cases of twins. Among twin pregnancies, conditions of monochorionic twinning, commonly regarded as monozygotic, are more complicated than dichorionic ones.

**Case summary:**

We systematically evaluated the cause of fetal death for a Han Chinese woman with monochorionic twinning following *in vitro* fertilization/embryo transfer. Discrepant karyotypes were unexpectedly discovered between the twins. One fetus had an aneuploid male karyotype (46, XY), dup (9) (p24.3-q13), and the other had a normal female karyotype (46, XX). We considered that the male died of aberration of chromosome 9 and the female died of subsequent acute exsanguination through vascular anastomosis.

**Conclusion:**

This study demonstrated the importance of recognizing the presence of monochorionic dizygotic twinning and the challenges of clinical management for twins following *in vitro* fertilization/double embryo transfer.

## 1. Introduction

Intrauterine fetal death (IUFD) not only impairs female fertility, but is an emotionally devastating event for family. When IUFD occurs, finding out the cause of fetal death has important clinical implications (1). Many causes of fetal death are known to recur in subsequent pregnancies, increasing the rates of another death associated with that cause (2). It is therefore necessary to determine the potential causes of fetal death in order to formulate prevention strategies for subsequent pregnancies in women with a history of adverse pregnancy outcomes (3). Moreover, determining the cause of fetal death can reduce the medical expenses in subsequent pregnancies (4). In addition, a clear cause of death facilitates emotional healing of the family from the IUFD event (1). However, relatively few cases of fetal deaths have clear causes of death due to diverse etiologies, unclear pathogenesis, limited services, and insufficient investigations (5).

Pregnancies through artificial reproductive techniques (ARTs), if the fetus dies, will be more devastating for the family because they are associated with higher expectation, greater cost, and more medical procedures than spontaneous conception (6). Most women undergoing *in vitro* fertilization/embryo transfer (IVF-ET) prefer the placement of more than one embryos with the hope of improving chance of pregnancy success, which has become a major driver of twin pregnancies (7). However, the risk of fetal death is substantially increased in twin gestation, notably in monochorionic (MC) twins (8, 9). Commonly, dichorionic (DC) twinning takes place in IVF-ET when more than one fertilized eggs are placed into the uterus (10). Nevertheless, many cases of monochorionic dizygotic (MCDZ) twins are now recognized in women undergoing IVF-ET, which complicates the identification of the cause of fetal death in twinning as well as limiting genetic counseling and challenging pregnancy management.

Here, we present a case of MC twinning after IVF-ET. At 12^+6^ weeks of gestation, phenotypic discrepancy between the twins was detected by prenatal ultrasonography. One week later, ultrasound demonstrated the intrauterine death of both fetuses. In the subsequent investigation on etiology, the twins were identified as dizygotic (DZ) ones, and the causes of death for each fetus were elucidated separately. The case highlights the importance of realizing the existence of MCDZ and the challenges to clinical management after *in vitro* fertilization/double-embryo transfer.

## 2. Case presentation

**FIGURE 1:**
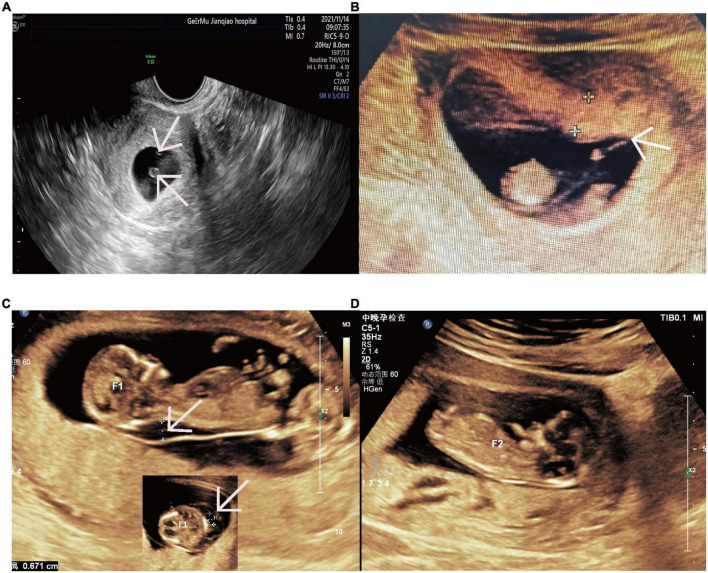
Ultrasound images revealing the monochorionic twins and twin A’s nuchal cystic hygromas and twin B’s normal NT. **(A)** Two yolk sacs within a single gestational sac at 6^+2^ weeks. The arrow indicates two yolk sacs. **(B)** The monochorionic diamnitic twins with a separating membrane of T sign at 10^+3^ weeks. The arrow indicates the distinct T sign of the separating membrane between the twins. **(C)** Nuchal cystic hygromas of twin A at 12^+3^ weeks. The arrows indicate the nuchal cystic hygromas of twin A from longitudinal section and transverse section. **(D)** The normal NT of twin B at 12^+3^ weeks. NT, nuchal translucency.

A 31-year-old woman of Han Chinese, gravida 2 para 0, had conceived *via* IVF-ET. Her first pregnancy at the age of 22, was artificially terminated in the first trimester because it was unwanted. The patient underwent bilateral endometrioma cystectomy at the age of 27. Subsequently, she thereafter had a diminished ovarian reserve with 0.40 ng/mL of anti-Mullerian hormone (AMH). A total of six eggs were obtained by two oocyte retrieval operations and were fertilized *in vitro*. After two failed double-embryo transfers, the remaining two embryos, respectively, retrieved in August 2020 and April 2021, were transferred after assisted hatching treatment. Her first ultrasonography at 6^+2^ weeks of gestation documented two yolk sacs within a single gestational sac (Figure 1A). The subsequent scan at 10^+3^ weeks of gestation demonstrated MC diamniotic twins with a T-shaped insertion of the membrane (Figure 1B).

Unfortunately, one of the twins (twin A) was revealed to have nuchal cystic hygroma and generalized hydrops without abnormal volume of amniotic fluid on ultrasonography at the time of routine nuchal transparency (NT) screening (Figure 1C). On the follow-up ultrasound, a structural malformation of the heart was observed. At the same time, no abnormalities were found on the other fetus (twin B) by detailed ultrasonic examination (Figure 1D). The couple underwent genetic counseling and the twins were interpreted as monozygosity due to the MC placenta, so they were informed of a high probability of genetic disorder or congenital anomaly for both twins. The couple intended to terminate the pregnancy accordingly.

After a week, at 13^+6^ weeks of gestation, both twins died spontaneously as the absences of cardiac activity were indicated on ultrasonography. Following a failed medical abortion, the patient then underwent a surgical curettage to terminate the pregnancy. During the fetal genetic examination, the twins were discovered to be inconsistent in their chromosomal composition. Twin A had a male karyotype with a partial trisomy 9, while twin B had a normal female karyotype. DNA zygosity studies of the twins demonstrated that the twins were dizygotic. A timeline of relevant events to the patient from conceiving to terminating pregnancy is shown in Table 1.

**TABLE 1:** A timeline of relevant events to the patient from conceiving to terminating pregnancy.

Date	Events	
August 11,2020	The first oocyte retrieval	Three eggs were retrieved and fertilized, then cultured for 3 days and cryopreserved.
December 30,2020	The first transplant	Two cleavage-stage embryos were transplanted.
April 17,2021	The second oocyte retrieval	Three eggs were retrieved and fertilized, then cultured for 3 days and cryopreserved.
July 17,2021	The second transplant	Two cleavage-stage embryos were transplanted.
September 3,2021	LMP	Last menstrual period.
October 18,2021	The third transplant	The two remaining embryos were transferred.
November14,2021	6 2/7 weeks’ gestation	Two yolk sacs within a single gestational sac were detected by transvaginal ultrasound.
November 19,2021	7 0/7 weeks’ gestation	Two germs with beating tube in a gestational sac were detected by transabdominal ultrasound.
December 13,2021	10 3/7 weeks’ gestation	Ultrasound demonstrated the monochorionic diamniotic twins.
December 27,2021	12 3/7 weeks’ gestation	One of the twins (twin A) was revealed to have nuchal cystic hygromas and generalized hydrops.
December 30,2021	12 6/7 weeks’ gestation	Structural malformation of the heart of twin A was observed on ultrasound.
January 6,2022	13 6/7 weeks’ gestation	Absence of cardiac activity of twins was indicated on ultrasonography.
January 13,2022	14 6/7 weeks’ gestation	The pregnancy was terminated by a surgical curettage.

## 3. Methods

### 3.1. Evaluation of maternal condition

The patient’s clinical history and medical records were reviewed in detail, including past medical history, pre-pregnancy and prenatal care. Clinical routine laboratory testing and additional detection were performed in hospital, including serological tests for immunodeficiency virus (HIV) and syphilis, thyroid-stimulating hormone (TSH), screening for blood group-related antibodies, lupus anticoagulant, and antiphospholipid antibodies.

### 3.2. Fetus and placenta examination

Fetal gross observations as well as placental macroscopic and microscopic histopathologic examination were performed according to standard procedures.

### 3.3. Genetic evaluation

Molecular karyotyping, chromosomal microarray analysis (CMA), was conducted using the Single Nucleotide Polymorphism (SNP) Array with CytoScan 750 K probes (Affymetrix, Santa Clara, CA, USA). At the same time, tissue samples of twin A, twin B and the placenta were stored at –80°C. High throughput next generation sequencing (NGS) was carried out on each cryopreserved specimen and analyzed using Sentieon software by Beikang Laboratory (Beikang Medical Laboratory Co, Ltd, Shandong, China). Short tandem repeat (STR) markers were detected on maternal and fetal DNA using the Human Identification Installation Kit (Applied Biosystems, Carlsbad, CA, USA) to monitor the maternal contamination.

### 3.4. DNA zygosity studies

The zygosity study was conducted on the data of NGS and STR from the tissue of each fetus and maternal blood. Sixteen STRs and AMEL-X markers were used to determine the relationship among the specimens. Identity-by-descent (IBD) was analyzed based on the combined SNP/InDel using King software.

## 4. Results

### 4.1. Evaluation of the maternal condition

The couple were not consanguineous and the woman’s medical history that associated with fetal death was unremarkable. She had neither been exposed to chemicals, drugs, smoking, alcohol, and viral infections, nor had she suffered any discomfort during pregnancy. Her body mass index (BMI) was 23. All laboratory values were unremarkable in the first trimester of pregnancy and during hospitalization, including negative tests for lupus anticoagulant, antiphospholipid, and antinuclear antibodies. Accordingly, underlying maternal contributors to fetal death were excluded.

### 4.2. Examination of fetus and placenta

**FIGURE 2:**
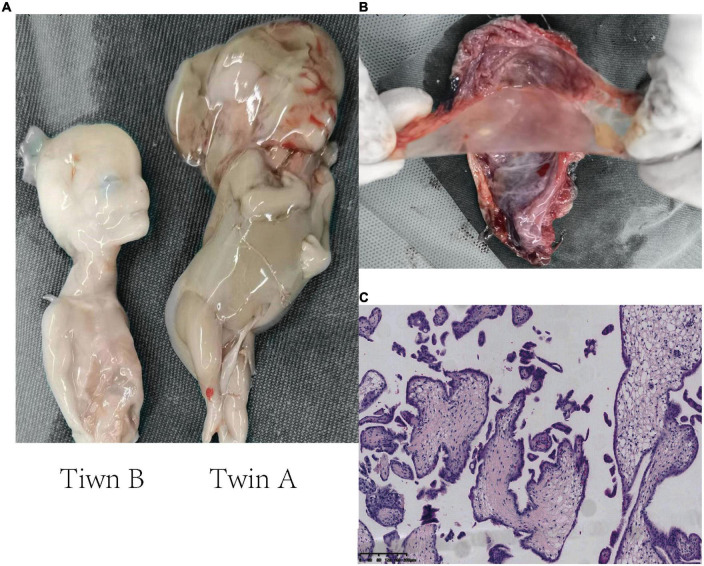
Histopathology images showing the examination of two fetuses and the placenta. **(A)** Macroscopic examination of the twins. Twin A (right), severe cystic hygroma and generalized hydropic foetalis, appearing decaying tissue. Twin B (left), unremarkable macroscopic abnormality. **(B)** The monochorionic diamniotic placenta of the twins. The placental septum indicates two amniotic sacs beside thin membranes. **(C)** Microscopic histopathological examination of the placental section (H&E staining, 10×).

The fetus (twin A) with nuchal cystic hygroma and generalized edema had been identified by macroscopic examination and appeared decaying tissue, while twin B was grossly unremarkable (Figure 2A), suggesting that twin A had died before twin B. Gross examination of the placenta confirmed that the twins were MC diamniotic with a thin separating membrane (Figure 2B). Microscopic examination of the placenta did not identify typical infarction, necrosis, vascular thrombosis, or inflammatory cell infiltration, which excluded that placental lesions were the cause of fetal death (Figure 2C).

### 4.3. Genetic evaluation

The CMA result of twin B showed a normal female karyotype (46, XX) without copy number variations (CNVs) or loss of heterozygosity (LOH). The NGS analysis of twin A showed an unbalanced male karyotype (46, XY) with dup (9) (p24.3-q13), resulting in partial trisomy 9. The duplicated region was interpreted as seq[GRCh 37/hg19] (10001-68350000) X3, including the whole short arm of chromosome 9, a 48.42 Mb fragment, involving 220 protein-coding genes, 49 of which were potentially pathogenic genes included in OMIM (Supplementary Figure 1A). A reanalysis of twin B by NGS confirmed the normal 46, XX karyotype (Supplementary Figure 1B). The NGS analysis for placental genetic make-up showed 46, XY with dup (9) (p24.3-q13), consistent with twin A (Supplementary Figure 1C).

### 4.4. Analysis of DNA zygosity

The zygosity analysis showed that 7 of 17 genetic markers were different between the twins. In the specimens of each fetus, there was at least one allele from the mother at all marker loci. The results were consistent with DZ twins (Table 2). IBD analysis yielded kinship value of 0.2754, indicating first-degree relatives of the twins [inference criteria: monozygotic twin < 0.1; full sibling 0.1–0.365 (11)].

**TABLE 2:** DNA zygosity analysis in samples from each fetus and the maternal peripheral-blood lymphocytes.

No.	STR marker	Chromosomes	Twin A alleles	Twin B alleles	Maternal alleles
1	Amelo	X/Y	X/Y	X/X	X/X
2	D13S317	13	A8/A12	A9/A9	A9/A12
3	D16S539	16	A9/A12	A9/A12	A12/A12
4	D18S51	18	A13/A13	A13/A13	A13/A13
5	D2S1338	2	A20/A23	A20/A25	A18/A20
6	D5S818	5	A11/A13	A11/A13	A11/A11
7	D7S820	7	A11/A11	A11/A12	A11/A12
8	D8S1179	8	A13/A14	A13/A16	A14/A16
9	D8S588	8	A12/A13	A12/A13	A8/A13
10	G10S0001	10	A14/A22	A14/A22	A14/A19
11	G15S0001	15	A11/A15	A11/A15	A11/A15
12	G2S0002	2	A21/A21.2	A20.2/A21.2	A21.2/A22.2
13	G4S0001	4	A17/A18	A17/A18	A15/A18
14	G5S0001	5	A7/A10	A7/A10	A7/A9
15	G7S0005	7	A12/A12	A9/A12	A10/A12
16	THO1	11	A9/A9	A9/A9	A9/A9
17	VWA	12	A17/A19	A17/A19	A17/A17

### 4.5. Counseling and formulating preventive strategies

A detailed etiological explanation and valid counseling were provided to the couple as follows: in their next pregnancy, preimplantation genetic diagnosis (PGD) for aneuploidy on their blastocysts is not necessary, but prenatal diagnostic procedure for aneuploidy and copy number variations through amniocentesis samples is recommend. The transfer of a single blastocyst can be chosen to reduce the risk of pregnancy complications.

## 5. Discussion

**FIGURE 3:**
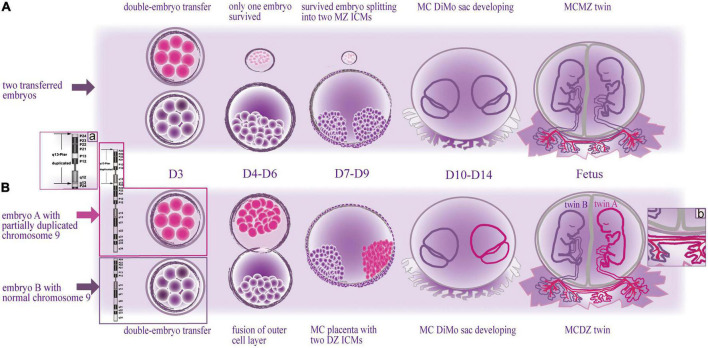
Schematic drawing of two types of embryonic development with monochorionic diamniotic twins after double-embryo transfer. (Above) The formation of monochorionic monozygotic twins in common cases. (Below) The formation of monochorionic dizygotic twins in this case. MZ, monozygotic; MC, monochorionic; DZ, dizygotic; DiMo, diamniotic; ICM, inner cell mass; D, day. **(A)** Enlarged view illustrates the cause of death of twin A, the duplicated segment of chromosome 9; **(B)** enlarged view illustrates the cause of death of twin B, the vascular anastomoses between the twins.

Our research indicates: First, the two fetuses are dizygosity, which can explain the phenotypic discrepancy between them whereas they were monochorionic (MC) twins. Twin A probably died of the aberration of chromosome 9 (Figure 3a). Second, twin A died prior to twin B. Due to the MC condition, twin B possibly died from the acute exsanguination caused by the death of twin A *via* vascular anastomosis of the placenta (Figure 3b) whereas they were DZ twins.

Chorion development is expected to take place on embryonic days 4–7 (D4-D7), and amnion starts to form between days 7 and 9 (D7-D9) (12). Based on literature and theory of embryology, we speculated on the mechanism of the monochorionic dizygotic (MCDZ) embryologic event of this case (Figure 3). The woman was transplanted with two cleavage-stage embryos cultured *in vitro* for 3 days (D3). If the two transferred embryos merged during the stage of D4-D7, they may potentially share a common chorionic membrane with separate amniotic membranes (10). The twins, initially, were judged to be monozygotic (MZ) as they had the MC placenta. Hence, the initial assumption was that only one of the transferred embryos survived and split into two (Figure 3 Above). Given that the twins were dizygotic (DZ), the fusion of the outer cells of the DZ embryos was speculated before implantation, while the DZ inner cells mass (ICM) developed into two fetuses with separate amniotic cavities, which resulted in the MC placenta of the DZ twins (Figure 3 Below) (13).

A case of MCDZ twinning is hard to be identified. Most of them were noted due to gender discordance on fetal anatomy survey by ultrasound or prenatal cell free fetal DNA screening (14, 15), and were further confirmed. It is not easily noted to the same gender. So, cases of MCDZ twins of *in vitro* fertilization/double-embryo transfer may be far more than those of what we know. However, prenatal determination of zygosity in MC twins is important for decision making when one of the twins is suspected of having a congenital anomaly (16). In this study, at first, DNA was extracted only from twin B for CMA detection as twin A’s tissue was decaying and dizygosity was not realized. Consequently, we made the conclusion with normal karyotypes of the twins. However, given the ultrasound abnormalities of twin A, a chromosomal disorder of the fetus was still suspected. Analyzing the DNA from the cryopreserved specimens, we discovered the imbalance of chromosome 9 at twin A, involving the duplication of the entire short arm and partial long arm (Figure 3a) as well as accidently discovered the non-identical karyotype between the MC twins.

Trisomy of the short arm of chromosome 9 (Trisomy 9p) is the fourth most common autosomal trisomy after 21, 18, and 13 trisomy (17). The severity of phenotype correlates with the extension of duplicated chromosomal segments (18). If the duplicated segments include the long arm of chromosome 9, clinical findings may resemble trisomy 9 mosaic syndrome which is associated with cardiac anomalies (19). The karyotypes of the parents were normal (Karyotype analysis during the couple’s planning for IVF-ET), indicating that the trisomy 9 of twin A was a *de novo* genetic change. We cannot identify the cause of chromosomal aberration in twin A whereas without abnormity in twin B. We suspected it might be related to the conditions of fertilization as the two eggs were retrieved and fertilized separately at different times.

Generally, DZ twins, which develops from two separate fertilized eggs, would be expected to be dichorionic (DC) (10), i.e., two fetuses with separate placentas. The blood between the two placentas does not communicate even if they appear to be fused together, which is likely harmless to the other if one of the twins dies (20). However, we confirmed that the DZ twins shared one placenta by the examination of the placenta (Figure 2). Nearly all MC twins are present vascular anastomosis, which is the primary pathology of adverse outcomes in MC twins (21). Following a single death of twins, blood volume can shift rapidly through the anastomosis from the living fetus toward the deceased co-twin. Due to acute exsanguination, the living fetus can subsequently suffer hypoperfusion, hypotension and anemia, which induced tissue hypoxia, acidosis and damage, culminated in the death of the second twin (22). So we speculated the possible death of twin B was exsanguination after death of twin A due to vascular anastomosis (Figure 3b). However, a fluid injection assay for testing vascular anastomosis failed to be achieved because of the rupture of the umbilical cord caused by the curettage and too thin umbilical cords due to small gestational age, through which can verify reliably the vascular anastomosis between the two fetuses.

The reason for the formation of MCDZ twins associated with IVF could possibly be due to simultaneous ET, in which two fertilized eggs are located in close proximity (23). In addition, assisted hatching techniques introduce small openings or thin areas in the zona pellucida, which may increase the likelihood of fusion (14).

Ignoring the existence of MCDZ can result in incorrect decision-making of the couple. In this case, prior to the spontaneous death of the twins, the couple had already decided to terminate the pregnancy because of improper counseling based on the MZ twins by mistake. In fact, not all fetuses with chromosome 9 abnormalities necessarily suffer IUFD (24). Without the death of twin A, further invasive investigations could have been performed by respective amniocentesis and selective feticide could have been a preferred approach after ultrasound-guided bipolar cord coagulation or radiofrequency ablation to induce complete circulatory confinement of the twins. It would be possible to obtain a normal fetus under such conditions.

One of the key points in our study was the cryopreservation of the fetal specimens. DNA analysis from the fetus provides a “molecular autopsy” to determine the cause of fetal death and is valuable for the diagnosis of many genetic diseases (25). Without the additional stored fetal specimens, this case would have been an unexplained one. Despite the thorough etiology examination, the patient would remain anxious and sought additional tests, unnecessary interventions and frequent visits in her next pregnancies. The American College of Obstetricians and Gynecologists (ACOG) recommended performing fetal genetic analysis after all cases of stillbirth (26). Here, we suggested it is necessary to store the fetal specimens when the genetic analysis cannot be performed right after IUFD.

Another essential part of our study was the timely investigation. Without delay, we reviewed the patient’s medical history. Following detailed observation at delivery, we recognized the abnormal twin A revealed by ultrasonography and verified that twin A died before twin B, which could help explain the death of twin B.

One limitation of our study is that the molecular detection of the placenta was not taken from multiple samples at different locations under each gestational sac. If such work had been done, the mosaicism of placenta might have been detected, which would have been helpful to explain the fusion mechanism of the early embryos.

The judgment that MC twins are necessarily MZ ones is dogmatic. It is important to realize the existence of MCDZ twinning after *in vitro* fertilization/double-embryo transfer. The specific formation of MCDZ should be further studied.

## Ethics statement

Ethical review and approval was not required for the study on human participants in accordance with the local legislation and institutional requirements. Written informed consent from the patients/participants or patients/participants legal guardian/next of kin was not required to participate in this study in accordance with the national legislation and the institutional requirements. This study was approved by the Institutional Ethics Committee (approval number: 2021-017) and informed consent was obtained from the patient for the publication of the clinical information.

## Author contributions

AX and YC conceived the study. AX drafted the initial manuscript and mapped the schematic drawing (Figure 3). GL and LT conducted the genetic evaluation. XC performed the ultrasound examination. XZ completed the pathological examination. JH collected the medical history of the patient. XW and YR revised the manuscript. All authors involved in the analysis of the data and determining the cause of the fetal death and approved the submitted version.

## Supplementary material

Supplementary Figure 1 Sequencing plot showing the inconsistency of genetic make-up between the twins as well as the placenta.
