# Supplementary figures and images for: Determining the cause of intrauterine fetal death in monochorionic twins: A case report
Source: Front Med (Lausanne). 2023 Jan 4;9:1055275. doi: 10.3389/fmed.2022.1055275 (PMC9846037; doi:10.3389/fmed.2022.1055275)

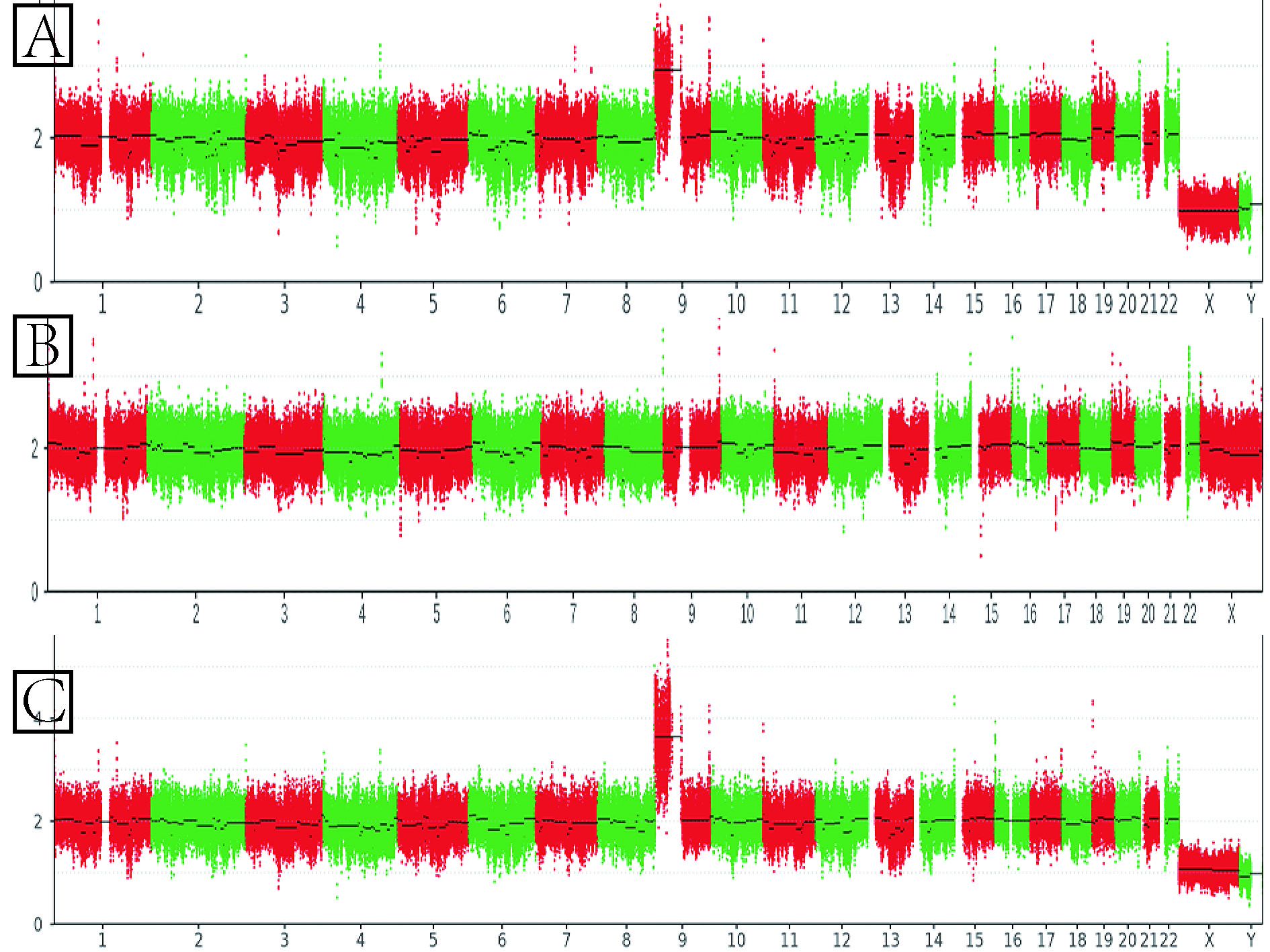

Supplement: Supplementary Figure 1 — Sequencing plot showing the inconsistency of genetic make-up between the twins as well as the placenta. [file Image_1.jpeg]
